# Evaluation of oral mucositis, candidiasis, and quality of life in patients with head and neck cancer treated with a hypofractionated or conventional radiotherapy protocol: a longitudinal, prospective, observational study

**DOI:** 10.1186/s13005-023-00356-3

**Published:** 2023-03-08

**Authors:** Pedro Maciel de Vasconcellos Ferreira, Marina de Castro Monteiro Franco Gomes, Ana Clara Speziali Menegazzi Almeida, Júlia Soares Cornélio, Thiago Jardim Arruda, Arnoldo Mafra, Marcelo Henrique Silva Nunes, Rafael Borges Salera, Raquel Fabiane Nogueira, Juliana Maria Braga Sclauser, Ana Paula Drummond-Lage, Bruno Almeida Rezende

**Affiliations:** 1 School of Medical Sciences of Minas Gerais, Alameda Ezequiel Dias, Belo Horizonte, 27530130-110 Brazil; 2 Mario Penna Institute, 901, Joaquim Candido Filho, Belo Horizonte, 30320-420 Brazil

**Keywords:** Oral Mucositis, Oral Candidiasis, Radiotherapy Dose Fractionations, Quality of Life, Radiation Dose Hypofractionation, Head and Neck Neoplasms

## Abstract

**Background:**

Due to the severe acute respiratory syndrome coronavirus 2 (SARS-CoV-2) pandemic, recently, Radiotherapy (RT) protocols requiring fewer sessions (hypofractionated) have been used to shorten RT treatment and minimize patient exposure to medical centers, and decrease the risk of SARS-CoV-2 infection.

**Methods:**

This longitudinal, prospective, observational study aimed to compare the quality of life (QoL) and the incidence of oral mucositis and candidiasis in 66 patients with head and neck cancer (HNC) who undergo a hypofractionated RT protocol (GHipo), total of 55 Gy for 4 weeks, or a conventional RT protocol (GConv), total of 66 − 70 Gy for 6 − 7 weeks.

**Purpose:**

To assess the incidence and severity of oral mucositis, the incidence of candidiasis, and QoL were evaluated using the World Health Organization scale, clinical evaluation, and the QLC-30 and H&N-35 questionnaires, respectively, at the beginning and the end of RT.

**Results:**

The incidence of candidiasis did not show differences between the two groups. However, at the end of RT, mucositis had a higher incidence (*p* < 0.01) and severity (*p* < 0.05) in GHipo. QoL was not markedly different between the two groups. Although mucositis worsened in patients treated with hypofractionated RT, QoL did not worsen for patients on this regimen.

**Conclusions:**

Our results open perspectives for the potential use of RT protocols for HNC with fewer sessions in conditions that require faster, cheaper, and more practical treatments.

**Supplementary Information:**

The online version contains supplementary material available at 10.1186/s13005-023-00356-3.

## Background

Head and neck cancer (HNC) affects approximately 500,000 individuals of both sexes annually worldwide and is the fifth most prevalent type of malignant tumor [1]. Radiotherapy (RT) is one of the treatments used to treat HNC in most cases and may be associated with chemotherapy and surgery [2]. The radiation dose required to achieve the best therapeutic index in the treatment of HNC depends on the type of tumor being treated and the nature of the normal tissues within the volume to be irradiated. The RT protocol most commonly used in Brazil (conventional RT protocol) is based on the administration of 5 weekly fractions of 1.8 − 2.0 Gy, with a total of 66 − 70 Gy for 6 − 7 weeks [3, 4]. However, other RT protocols for HNC have been proposed for therapeutic individualization to provide alternatives with better outcomes in cancer treatment. During the coronavirus disease 2019 (COVID-19) pandemic, different protocols were designed to increase the practicality of radioactive therapy, reduce costs, or even minimize adverse effects [5, 6].

One of the alternatives to the conventional protocol is a protocol that involves hyperfractionation, which consists of delivering small fractions of radiation twice a day. The total radiation dose may be the same as that observed in the conventional protocol or higher. Conversely, protocols modified by reducing the number of sessions, commonly called hypofractionated or hypofractionation, are not very widespread. In these protocols, the total radiation dose is divided into large doses (> 2.0 Gy per dose) and the treatments are administered once a day for a shorter period (fewer days or weeks) compared to the conventional protocol [7]. However, according to Huang et al. [8], unlike hyperfractionation protocols, hypofractionation protocols are not addressed in meta-analyses of RT in HNC, and there are no randomized phase III clinical trials to extract data that confirm their efficacy for controlling these types of tumors.

RT treatment of HNC causes both short- and long-term side effects in the oral cavity. The most common side effects in the oral cavity are mucositis, candidiasis, hyposalivation, and changes in swallowing. Mucositis is a condition characterized by an inflammatory reaction in the digestive tract and oral cavity due to scaling and limited cell renewal of the mucosa [9]. Its etiology is complex and may be related to hyposalivation [9, 10]. In cancer patients, this condition can cause long-term swallowing impairments, contributes to low adherence/ interruption of RT treatment, and provide a negative impact on the quality of life and survival time [10]. Treatment of mucositis involves low-level laser therapy and topical use of anti-inflammatory solutions [11]. However, it is not always satisfactory, unlike other conditions which are easier to manage, such as candidiasis, characterized by infection caused by the fungus *Candida albicans* that can develop in the mucous membranes of the mouth after alterations in the oral microbiota [12].

The emergence of the novel COVID-19 pandemic at the end of 2019 brought several societal changes. The situation required a rapid response from governmental entities to create mechanisms to reduce society’s exposure to conditions that predispose to infection with severe acute respiratory syndrome coronavirus-2 (SARS-CoV-2). Recent data suggest that cancer patients are at higher risk of being infected with SARS-CoV-2 than the general population and, if infected due to immune weakness, generally have worse disease progression [13–15]. These risks can be mitigated by adopting treatment strategies that reduce the number of hospital visits for systemic chemotherapy and/or RT sessions, decreasing the risk of SARS-CoV-2 infection.

Several entities have recommended that the hypofractionation technique be applied whenever possible to minimize the exposure of cancer patients to situations predisposing them to infection with SARS-CoV-2 [16, 17]. Therefore, the pandemic was an opportunity to study the hypofractionation protocol, which has limited literature data about the treatment of HNC [6]. A hypofractionation protocol was used as an alternative to the conventional protocol during the COVID-19 pandemic at the Mario Pena Institute in Belo Horizonte, a reference center for RT in patients with HNC. Therefore, the objective of this study was to evaluate the incidence of mucositis, the incidence of candidiasis, and the quality of life (QoL) in patients with HNC treated with conventional or hypofractionated RT and compare the results obtained for the two RT protocols.

## Methods

### Study design and participants

This longitudinal, prospective, observational study was conducted in the outpatient support clinic for patients undergoing cancer treatment at the Mario Penna Institute (Belo Horizonte, Minas Gerais, Brazil). All patients assisted between March to December 2021 were invited to participate in the study. This period covers the whole use of hypofractionated RT protocol in the institution.

Participants of both sexes, aged > 18 years with histopathological confirmation of squamous cell carcinoma in the oral cavity, larynx, pharynx, maxillary sinus, and salivary glands with an indication of conventional or hypofractionated RT were included in the study. Exclusion criteria were as follows: patients who continued to consume alcohol and tobacco during RT treatment, patients with an RT regimen different from the study protocols, patients who used a nasogastric tube before RT treatment, and patients who were clinically unable to open their mouths for intraoral evaluation.

A total of 109 patients were included in the study and stratified into two groups:

- Conventional protocol group (GConv): Initially composed of 73 participants who underwent a conventional RT protocol consisting of the administration of 5 weekly fractions of 1.8 − 2.0 Gy, with a total of 66 − 70 Gy for 6 − 7 weeks.
- Hypofractionated protocol group (GHipo): Initially composed of 36 patients who underwent an RT protocol consisting of the administration of 5 weekly fractions of 2.75 Gy, with a total of 55 Gy for 4 weeks.

For a short period of time, at the most critical moment of the COVID-19 pandemic in Brazil, the Institution implemented the Hypofractionated RT protocol following the recommendations of international guidelines or recent works[16, 17]. Patients belonging to the GHIpo group were selected during this period. After the suspension of the indication of the hypofractionation radiotherapy protocol, a convenient sample was selected from patients submitted to the conventional RT protocol to create a control group (GConv).

Of the total number of participants included, 66 completed all stages of the study, totaling 23 participants for GHipo and 43 for GConv. The remaining participants were excluded due to death, interruption of treatment, refusal to continue in the study, or noncompliance with all stages proposed in the study. The sample calculation considered a prevalence of mucosistis of 61%[18, 19], with 95% confidence. Therefore, the sample would have 92 subjects and considering the loss of up to 30%, the final sample of 66 patients is acceptable.

### Oral evaluation of mucositis and candidiasis

The presence and severity of oral mucositis and candidiasis were assessed in all participants at three-time points of the study: i) on the day of the first RT session (initial assessment); ii) halfway through RT treatment (intermediate assessment); and iii) on the day of the last RT session (final assessment). The intermediate evaluation occurred between the 14^th^ and 20^th^ RT sessions for the participants assigned to the GConv and between the 8^th^ and 12^th^ sessions for the GHipo.

Oral mucositis was evaluated according to the methodology proposed by the World Health Organization (WHO), which established the classification criteria to assess this oral mucosa condition [20]. It is a visual and noninvasive evaluation of the oral cavity, where the categorization follows the following parameters: grade 0, no changes; grade 1, pain/erythema; grade 2, erythema and ulcers; grade 3, ulcers (liquid diet only); grade 4, it is not possible to ingest food.

The presence of pseudomembranous candidiasis was clinically evaluated [21]. This evaluation was based on the observation of anatomic localization, features, and removal of the pseudomembrane by rubbing the lesion with gauze, with the adjacent mucosa appearing normal or erythematous, usually painless [21].

Both evaluations were performed on all participants by a single operator with extensive academic training in dentistry and clinical experience, properly trained in all stages proposed to minimize biases related to different perceptions of the oral condition of the participants. In addition to visual evaluation, the participant was also asked about the intensity of pain and difficulty swallowing food.

### QoL assessment

QoL was assessed using the questionnaires recommended by the European Organization for Research and Treatment of Cancer, the QLQ-C30 (version 3.0), and QLQ-H&N35 validated for Portuguese [22, 23]. The first assesses the symptoms and QoL of cancer patients in general. It comprises 30 items distributed on scales that measure symptoms and functional aspects commonly related to cancer. Higher scores on the symptom scale represent more/worse symptoms. Conversely, higher scores on the functional scales represent a higher ("better") QoL or a higher level of functioning [24]. The QLQ-H&N35 is a specific module for patients with HNC that must be applied together with the QLQ-C30 questionnaire [23]. This module allows the evaluation of seven specific domains of HNC, namely, pain, swallowing, senses (taste and smell), speech, social eating, social contact, and sexuality. In addition to these, there are 11 specific items on problems, such as dental, trismus, xerostomia, thick saliva, cough, malaise, use of analgesics, nutritional supplements, feeding tubes, and weight loss or gain. Both have items on a 4-point Likert scale (i.e., not at all, 1 point; little, 2 points; moderate, 3 points; and very much, 4 points). The QLQ-C30 questionnaire also has two items on the perception of QoL and health on a 7-point scale (1, very poor; 7, excellent). All scales in both questionnaires are represented by scores ranging from 0 to 100.

The patients answered the two questionnaires in the clinic at two different moments, before the first and after the last RT sessions.

### Clinical data and lifestyle research

The patients responded to a structured questionnaire prepared by the researchers to evaluate clinical and sociodemographic conditions and habits such as smoking, alcoholism, and oral hygiene. This questionnaire was applied by the researchers. In addition, patients' medical records were analyzed to collect information related to general health, pre-existing pathologies, medications used, tumor staging and location, and RT and chemotherapy protocols used.

### Statistical analysis

Exploratory statistical techniques were used to analyze the data, which allowed for better visualization of the general characteristics of the data. Data were presented in frequency tables with absolute frequencies and their respective percentages and descriptive measures (mean, median, standard deviation, minimum and maximum) for quantitative data. Quantitative variables were tested for normality by the Kolmogorov − Smirnov test. As the continuous variables referring to the QoL questionnaires did not present a normal distribution, non-parametric tests were used (Mann − Whitney test and Wilcoxon test). The categorical variables were compared using the chi-square test, and when they presented expected frequencies below 5, the Fisher's test and the mesh-based Monte Carlo simulation were used for more than two response categories. The significance level adopted in all tests was 5%, therefore, the comparisons with a *p*-value lower than or equal to 5% were considered significant. The software used for the analyses was SPSS version 25.0.

## Results

The mean age of the 66 patients was 60.8 years (± 11.1), 65 years (± 10.8) for the GHipo, and 59 years (± 10.8) for the GConv. The groups were homogeneous in terms of sex, race, and marital status. Pathology was also similar in both groups, and patients had a low proportion of comorbidities, except for arterial hypertension, representing 34.8% of the total sample. Regarding drinking and smoking habits, GHipo presented a higher proportion of former smokers (73.9%) than GConv (46.5%), but a lower proportion of patients who had never smoked (*p* = 0.048). Regarding alcohol consumption, the groups did not differ (Table 1).

**Table 1:** Sociodemographic and dental characteristics, pathologies, and habits of the patients studied

Variables	Group			
	GHipo (*n* = 23)	GConv (*n* = 43)	Total (*n* = 66)	*p*-value
**Age** (mean [SD])	64.8 (10.8)	59.6 (10.7)	60.9 (11.1)	0.040
**Sex**	n (%)	n (%)	n (%)	
Female	4 (17.4%)	11 (25.6%)	15 (22.7%)	0.449^#^
Male	19 (82.6%)	32 (74.4%)	51 (77.3%)	
**Race**				
Pheoderma	14 (60.9%)	30 (69.8%)	44 (66.7%)	
Leukoderma	7 (30.4%)	9 (20.9%)	16 (24.2%)	0.784^#^
Melanoderma	2 (8.7%)	4 (9.3%)	6 (9.1%)	
**Marital Status**				
Married	12 (52.2%)	21 (48.8%)	33 (50.0%)	
Divorced	2 (8.7%)	5 (11.6%)	7 (10.6%)	0.413^#^
Single	4 (17.4%)	13 (30.2%)	17 (25.8%)	
Widower	5 (21.7%)	4 (9.3%)	9 (13.6%)	
**Diabetes**				
No	21 (91.3%)	40 (93.0%)	61 (92.4%)	1.000^#^
Yes	2 (8.7%)	3 (7.0%)	5 (7.6%)	
**Hypertension**				
No	13 (56.5%)	30 (69.8%)	43 (65.2%)	0.282^#^
Yes	10 (43.5%)	13 (30.2%)	23 (34.8%)	
**Respiratory disease**				
No	22 (95.7%)	40 (95.2%)	62 (95.4%)	1.000^# # #^
Yes	1 (4.3%)	2 (4.8%)	3 (4.6%)	
**Gastrointestinal disease**				
No	22 (95.7%)	40 (95.2%)	62 (95.4%)	1.000^# # #^
Yes	1 (4.3%)	2 (4.8%)	3 (4.6%)	
**Neurological disease**				
No	20 (87.0%)	37 (88.1%)	57 (87.7%)	1.000^#^
Yes	3 (13.0%)	5 (11.9%)	8 (12.3%)	
**Rheumatic disease**				
No	23 (100.0%)	40 (95.2%)	63 (96.9%)	0.536^# # #^
Yes	0 (0.0%)	2(4.8%)	2 (3.1%)	
**Smoking**				
Never smoked	1 (4.3%)	10 (23.3%)	11 (16.7%)	0.050^# #^
Smoker	5 (21.7%)	13 (30.2%)	18 (27.3%)	
Former smoker	17 (73.9%)	20 (46.5%)	37 (56.1%)	
**Drinker**				
No	14 (60.9%)	25 (58.1%)	39 (59.1%)	0.830^#^
Yes	9 (39.1%)	18 (41.9%)	27 (40.9%)	

*SD* Standard deviation, *GHipo* Hypofractionation group, *GConv* Conventional group

^#^Chi-square test

^# #^Monte Carlo simulation

^# # #^Fisher's test

Table 2 presents a comparison of tumor-related variables, as well as the type of treatment according to the groups evaluated. The most frequent tumor location was the oral cavity for both groups, and the distribution of the other locations was also similar (*p* = 0.779). The patients had more advanced disease stages (stages III and IV) in both groups (*p* = 0.465), and most underwent concurrent chemotherapy in the groups evaluated (*p* = 0.611). The medians for the number of RT sessions indicate the treatment plan that defines patients as belonging to GHipo or GConv (*p* < 0.001) (Table 2).

**Table 2:** Tumor characteristics and type of treatment

Variables	Group			
	GHipo (*n* = 23)	GConv (*n* = 43)	Total (*n* = 66)	*p*-value
**Primary tumor**	n (%)	n (%)	n (%)	
Oral cavity	12 (52.2%)	16 (37.2%)	28 (42.4%)	0.779^#^
Pharynx	7 (30,4%)	16 (37.2%)	23 (34.8%)	
Salivary gland	2 (8.7%)	3 (7.0%)	4 (7.6%)	
Larynx	1 (4.3%)	5 (11.6%)	6 (9,1%)	
Maxillary sinus	1 (4.3%)	3 (7.0%)	4 (6.1%)	
**Clinical stage**^**a**^				
Early (0, I, II)	2 (8.7%)	8 (18.6%)	10 (15.1%)	0.465^# #^
Advanced (III and IV)	21 (91.3%)	34 (79.1%)	55 (25.8%)	
**Concomitant chemotherapy**				
No	9 (39.1%)	20 (46.5%)	29 (43.9%)	0.611^# #^
Yes	14 (60.9%)	23 (53.5%)	37 (56.1%)	
**Radiotherapy sessions** Median (Min–Max)	20 (20–20)	35 (29—35)		

GHipo, Hypofractionation group; GConv, Conventional group

^a^1 Patient in GConv without Clinical stage information

^#^ Monte Carlo simulation

^# #^ Chi-square test

The incidence of pseudomembranous candidiasis in the intermediate and final evaluations was not statistically significant concerning the type of RT treatment performed in the study patients (*p* = 0.656 and *p* = 0.730, respectively). However, the evaluation of the mucositis showed a significant difference, with a higher severity of mucositis for GHipo in the initial and final evaluation (*p* < 0.05) (Table 3). In the initial evaluation, no patient had clinical signs of mucositis or candidiasis (data not shown). Only one patient in the study, belonging to Ghipo, had grade 4 mucositis (data not shown).

**Table 3:** Evaluation of candidiasis and mucositis in the groups studied

Variables	Group			
	GHipo (*n* = 23)	Gconv (*n* = 43)	Total (*n* = 66)	*p*-value
**Candidiasis** **(Intermediate evaluation)**	n (%)	n (%)	n (%)	
No	22 (95.7%)	38 (88.4%)	60 (90.9%)	0.656^#^
Yes	1 (4.3%)	5 (11.6%)	6 (9.1%)	
**Candidiasis** **(Final evaluation)**				
No	19 (82.6%)	37 (86.0%)	56 (84.8%)	0.730^# #^
Yes	4 (17.4%)	6 (14%)	10 (15.2%)	
**Mucositis** **(Intermediate evaluation)**	n (%)	n (%)	n (%)	
No	9 (39.1%)	25 (58.1%)	34 (51.5%)	0.141^# #^
Yes	14 (60.9%)	18 (41.9%)	32 (48.5%)	
**Grade of mucositis** **(Intermediate evaluation)** Non-detectable (grade 0)	9 (39,1%)	25 (58,1%)	34 (51,5%)	
1 and 2	10 (43,5%)	18 (41,8%)	28 (42,4%%)	** < 0.05**^#^
3 and 4	4 (17,4%)	0 (0%)	4 (6,1%)	
**Grade of mucositis** **(Final evaluation)** Non-detectable (grade 0)	**6 (26,1%)**	27 (62,8%)	33 (50,0%)	
1 and 2	11 (47,8%)	15 (34,9%)	26 (39,4%)	** < 0.05**^#^
3 and 4	6 (26,1%)	1 (2.3%)	7 (10,6%)	

*GHipo* Hypofractionation group, *GConv* Conventional group

^#^ Fisher's test

^#^^#^Chi-square test

After stratifying the patients according to the use of concomitant chemotherapy, no statistical difference was observed between the two groups (*p* ≥ 0.05) (Table 1S).

The overall QoL of patients before and after RT treatment was compared in the two groups using the QLQ-C30 questionnaire. In the final assessment of the quality of life, the domains remained similar except for Fatigue, which had a higher score for GHipo (*p* < 0.05). Insomnia, which was statistically different in the initial assessment (*P* < 0.01), did not remain significant in the final assessment (*p* = 0.489) (Table 4).

**Table 4:** Evaluation of the quality of life by QLC-C30 for each group before and after the radiotherapy treatment

**QLQ-C30 domains**	**GHipo**						**GConv**						
	n	Mean	SD	Median	Min	Max	n	Mean	SD	Median	Min	Max	*p*-value^#^
Global health scale	22	65,5	22,8	66,7	0,0	100,0	43	74,4	18,9	75,0	8,3	100,0	0,071
Functional scales	22	72,8	17,6	68,9	33,3	100,0	43	80,3	15,6	82,2	40,0	100,0	0,100
Symptom scales	22	24,1	18,3	23,1	0,0	76,9	43	19,3	17,6	12,8	0,0	64,1	0,204
Physical function	22	77,6	25,7	86,7	0,0	100,0	43	85,8	20,2	93,3	0,0	100,0	0,113
Emotional function	22	50,8	34,1	58,3	0,0	100,0	43	64,6	29,4	66,7	0,0	100,0	0,115
Cognitive function	22	78,8	25,8	91,7	16,7	100,0	43	87,6	26,2	100,0	0,0	116,7	0,078
Social function	22	87,1	25,7	100,0	0,0	100,0	43	87,7	20,9	100,0	0,0	100,0	0,627
Performance of activities	22	84,9	29,5	100,0	0,0	100,0	43	83,3	30,0	100,0	0,0	100,0	0,809
Fatigue	22	24,2	25,3	16,7	0,0	77,8	43	21,7	23,9	11,1	0,0	77,8	0,653
Nausea and vomiting	22	7,6	22,3	0,0	0,0	100,0	43	9,1	23,1	0,0	0,0	100,0	0,657
Pain	22	39,4	38,0	33,3	0,0	100,0	43	34,2	35,2	33,3	0,0	100,0	0,603
Dyspnea	22	13,6	28,5	0,0	0,0	100,0	43	8,7	26,3	0,0	0,0	100,0	0,384
Insomnia	22	43,9	41,6	33,3	0,0	100,0	43	19,2	33,5	0,0	0,0	100,0	** < 0.01**
Loss of appetite	22	19,7	32,0	0,0	0,0	100,0	43	15,1	31,9	0,0	0,0	100,0	0,328
Constipation	22	27,3	39,4	0,0	0,0	100,0	43	12,0	25,0	0,0	0,0	100,0	0,192
Diarrhea	22	9,1	21,0	0,0	0,0	66,7	43	11,8	28,9	0,0	0,0	100,0	0,902
Financial difficulties	22	33,3	39,8	16,7	0,0	100,0	43	31,8	41,8	0,0	0,0	100,0	0,772
Global health scale	20	72,9	19,7	70,8	33,3	100,0	41	78,5	19,1	83,3	16,7	100,0	0,263
Functional scales	20	63,7	23,9	65,6	20,0	100,0	41	75,1	21,6	80,0	20,0	100,0	0,086
Symptom scales	20	33,5	16,9	32,1	2,6	61,5	41	27,0	17,9	25,6	2,6	74,4	0,128
Physical function	20	62,3	32,2	63,3	0,0	100,0	41	77,7	23,7	86,7	20,0	100,0	0,073
Emotional function	20	52,9	36,5	62,5	0,0	100,0	41	63,0	34,2	75,0	0,0	100,0	0,248
Cognitive function	20	74,2	32,7	100,0	0,0	100,0	41	80,8	28,0	100,0	0,0	100,0	0,627
Social function	20	81,7	27,5	100,0	0,0	100,0	41	90,3	19,3	100,0	16,7	100,0	0,219
Performance of activities	20	60,0	43,4	83,3	0,0	100,0	41	71,6	36,0	83,3	0,0	100,0	0,262
Fatigue	20	50,6	30,7	55,6	0,0	100,0	41	33,2	27,7	22,2	0,0	100,0	** < 0.05**
Nausea and vomiting	20	20,0	23,9	16,7	0,0	66,7	41	20,8	29,8	0,0	0,0	100,0	0,766
Pain	20	33,3	27,0	33,3	0,0	100,0	41	32,1	29,4	33,3	0,0	100,0	0,689
Dyspnea	20	16,7	27,6	0,0	0,0	66,7	41	11,7	25,3	0,0	0,0	100,0	0,476
Insomnia	20	35,0	41,1	16,7	0,0	100,0	41	25,1	32,3	0,0	0,0	100,0	0,489
Loss of appetite	20	41,7	44,4	33,3	0,0	100,0	40	42,7	44,6	33,3	0,0	100,0	0,927
Constipation	20	36,7	41,7	16,7	0,0	100,0	41	25,1	33,1	0,0	0,0	100,0	0,384
Diarrhea	20	11,7	27,1	0,0	0,0	100,0	41	6,7	20,0	0,0	0,0	100,0	0,551
Financial difficulties	20	35,0	41,1	16,7	0,0	100,0	41	35,8	41,8	33,3	0,0	100,0	0,908

*GHipo* Hypofractionation group, *GCon* Conventional group

^**#**^Mann–Whitney’s test

The HNC-specific QoL was assessed using the H&N-35 questionnaire, and the two groups were also compared. In the initial assessment, we observed that the scores of the domains “Feeling unwell and weight loss” were statistically higher in the GHipo (both with *p* < 0.01). In the final evaluation, only the “Cough” domain showed a statistically significant difference, with a higher score for GHipo (*p* < 0.05) (Table 5).

**Table 5:** Evaluation of the quality of life by H&N-35 for each group before and after the radiotherapy treatment

H&N-35 domains	**GHipo**						**GConv**						
	n	Mean	SD	Median	Min	Max	n	Mean	SD	Median	Min	Max	*p*-value^#^
**Initial evaluation**													
Pain	23	21.2	29.4	8.3	0.0	83.3	43	19.6	22.0	8.3	0.0	75.0	0.727
Swallowing	23	33.0	32.7	20.8	0.0	100.0	43	17.5	20.2	8.3	0.0	66.7	0.076
Sensory problems	23	9.8	19.0	0.0	0.0	83.3	43	11.1	24.6	0.0	0.0	100.0	0.350
Speech problems	23	36.4	35.8	38.9	0.0	100.0	43	28.9	33.1	11.1	0.0	100.0	0.467
Social eating problems	23	18.7	21.4	4.2	0.0	50.0	43	10.4	17.3	0.0	0.0	66.7	0.193
Social contact problems	23	17.9	23.8	13.3	0.0	86.7	43	10.7	18.7	0.0	0.0	66.7	0.051
Low sex drive	23	45.2	46.9	33.3	0.0	100.0	43	24.9	38.3	0.0	0.0	100.0	0.082
Changes in teeth	23	19.3	35.7	0.0	0.0	100.0	43	17.1	35.3	0.0	0.0	100.0	0.785
Trismus	23	27.3	40.7	0.0	0.0	100.0	43	20.7	34.8	0.0	0.0	100.0	0.621
Xerostomia	23	17.5	34.3	0.0	0.0	100.0	43	24.6	37.8	0.0	0.0	100.0	0.340
Thick saliva	23	48.5	47.9	50.0	0.0	100.0	43	33.4	39.8	33.3	0.0	100.0	0.304
Cough	23	34.8	37.8	33.3	0.0	100.0	43	21.5	33.2	0.0	0.0	100.0	0.138
Feeling unwell	23	51.5	46.8	66.7	0.0	100.0	43	20.0	35.7	0.0	0.0	100.0	** < 0.01**
Use of analgesics	23	59.1	50.3	100.0	0.0	100.0	43	57.2	49.5	100.0	0.0	100.0	0.860
Nutritional supplements	23	31.8	47.7	0.0	0.0	100.0	43	23.9	42.6	0.0	0.0	100.0	0.556
Tube feeding	23	27.3	45.6	0.0	0.0	100.0	43	7.3	25.8	0.0	0.0	100.0	0.051
Weight loss	23	77.3	42.9	100.0	0.0	100.0	43	43.2	49.5	0.0	0.0	100.0	** < 0.01**
Weight gain	23	22.7	42.9	0.0	0.0	100.0	43	30.8	46.2	0.0	0.0	100.0	0.440
**Final evaluation**													
Pain	23	47.1	27.2	50.0	0.0	100.0	43	40.5	25.6	33.3	0.0	91.7	0.334
Swallowing	23	52.1	37.8	37.5	0.0	100.0	43	39.8	27.8	33.3	0.0	91.7	0.254
Sensory problems	23	32.5	39.5	8.3	0.0	100.0	43	41.2	31.0	50.0	0.0	100.0	0.265
Speech problems	23	44.2	38.9	33.3	0.0	100.0	43	30.1	31.5	22.2	0.0	100.0	0.207
Social eating problems	23	39.5	27.5	41.7	0.0	100.0	43	31.9	29.2	33.3	0.0	100.0	0.278
Social contact problems	23	16.0	15.7	16.7	0.0	46.7	43	13.4	21.5	0.0	0.0	73.3	0.192
Low sex drive	23	49.1	43.6	66.7	0.0	100.0	43	41.1	43.3	33.3	0.0	100.0	0.526
Changes in teeth	23	9.8	22.9	0.0	0.0	66.7	43	13.3	30.0	0.0	33.3	100.0	0.695
Trismus	23	28.3	40.9	0.0	0.0	100.0	43	29.2	41.0	0.0	0.0	100.0	0.979
Xerostomia	23	38.3	43.6	33.3	0.0	100.0	43	59.0	42.5	66.7	0.0	100.0	0.102
Thick saliva	23	78.3	32.9	100.0	0.0	100.0	43	84.1	30.7	100.0	0.0	100.0	0.486
Cough	23	48.3	39.7	33.3	0.0	100.0	43	27.7	35.7	0.0	0.0	100.0	** < 0.05**
Feeling unwell	23	33.3	44.6	0.0	0.0	100.0	43	33.3	38.7	33.3	0.0	100.0	0.696
Use of analgesics	23	65.0	48.9	100.0	0.0	100.0	43	65.0	47.7	100.0	0.0	100.0	0.956
Nutritional supplements	23	80.0	41.0	100.0	0.0	100.0	43	72.6	44.7	100.0	0.0	100.0	0.469
Tube feeding	23	40.0	50.3	0.0	0.0	100.0	43	17.7	38.0	0.0	0.0	100.0	0.077
Weight loss	23	90.0	30.8	100.0	0.0	100.0	43	77.6	41.8	100.0	0.0	100.0	0.196
Weight gain	23	10.0	30.8	0.0	0.0	100.0	43	15.0	35.7	0.0	0.0	100.0	0.484

*GHipo* Hypofractionation group, *GCon* Conventional group

^**#**^Mann–Whitney’s test

## Discussion

The presented study proposed to evaluate the most prevalent acute side effects of RT in the treatment of HNC using two different RT protocols. Our data reveal that patients undergoing the hypofractionated protocol had a higher incidence and severity of oral mucositis. Despite this, they did not show important changes in the QoL. The hypofractionated protocol is rarely used as a curative treatment of HNC, as some studies suggest greater side effects of therapy, although many studies suggest similar efficacy to conventional protocols. However, studies such as Veluthattil et al. analyzed the grade of oral mucositis after 15 RT sessions (52.5 Gy) as a palliative treatment for HNC and showed that 72% of the patients had grade 3 mucositis, suggesting a high incidence of mucositis in protocols with higher radiation doses [25]. However, studies that evaluate the side effects of different hypofractionated RT protocols for curative purposes are scarce. To our knowledge, no other study has compared the incidence of oral mucositis and candidiasis, as well as QoL, between conventional and hypofractionated protocols.

Our study included patients with the same eligibility criteria in the homogeneous groups to avoid possible bias when comparing the different protocols. In addition to the homogeneity of the groups regarding clinical characteristics, the groups were also homogeneous regarding the type, location, and stage of the tumor. Chemotherapy is commonly applied concomitantly with RT in HNC treatment [1]. In our study, most of the participants received chemotherapy during the study. It is already described in the literature that most chemotherapeutic agents used in the treatment of HNC have the potential to cause short-term oral changes, especially mucositis [26]. Therefore, we also investigated whether there was an association between the incidence of mucositis and candidiasis and chemotherapy concomitant with RT. However, in our study, patients who received chemotherapy concomitant with RT did not show a higher incidence of these oral changes (Table 1S). Furthermore, the groups did not show differences in the use of chemotherapy associated with the RT protocols evaluated.

Candidiasis is usually present in patients undergoing RT for HNC due to immunosuppressive status, induced hyposalivation, and mucositis that leads to difficulties in establishing adequate oral hygiene and changes in the oral microbiota [27]. The most common form of candidiasis found in patients with HNC undergoing RT is pseudomembranous candidiasis, followed by erythematous candidiasis, while hyperplastic candidiasis is rarely reported [28, 29]. We found no studies in the literature that show a correlation between higher radiation doses and a higher incidence of pseudomembranous candidiasis. There were no differences in the incidence of candidiasis in the different stages evaluated for the two groups in our study. Lower rates of pseudomembranous candidiasis were found in our study population compared to those reported in other longitudinal studies evaluating the onset of candidiasis during RT treatment of HNC, such as that of Jham et al. [30]. A previous study by Chitapanarux et al. showed that the clinical and symptom-based analysis of oral candidiasis in irradiated patients with HNC might underestimate the diagnosis and that microbiological analysis may contribute to a higher number of positive diagnoses of this condition [31]. Our study only performed a clinical evaluation of oral candidiasis and not a microbiological analysis, which could explain the differences found since studies showing a high prevalence of this condition in irradiated patients used microbiological analysis to establish the diagnosis.

Oral mucositis occurred at a rate close to 50% at the end of the treatment and in the intermediate evaluation, which was performed approximately halfway through the RT protocol. Various studies have indicated an incidence of oral mucositis between 50 and 80% during conventional RT treatment, corroborating our findings for the total population of the present study [32, 33]. However, when separating the groups to evaluate the incidence of oral mucositis in different RT protocols, GHipo presented a higher percentage of patients with this condition at the end of the RT treatment. This was expected since some studies, mainly studies that used hypofractionated protocols for palliative treatment of HNC, have already suggested that changes in fractionation could predispose to a higher prevalence of mucositis [25, 32]. When evaluating the incidence of mild mucositis (grades 1 or 2) or more severe mucositis (grades 3 and 4) in the intermediate and final stages of the study, a worsening of the evolution of mucositis was observed in GHipo patients. Our data are similar to the results of a previous study by Veluthattil et al. that found 72% of HNC patients with grade 3 mucositis at the end of their hypofractionated RT treatment [25]. However, it should be noted that our hypofractionated protocol differs from the protocol used in the study by Veluthattil et al. since our study evaluates mucositis in curative RT treatment and is not exclusively palliative, as described by those authors. Another point to be discussed is that GHipo patients had a higher mean age. However, it is unlikely that this fact may be related to a higher incidence of oral mucositis in this group since there are numerous studies in the literature showing that age does not correlate with an increased risk of oral mucositis or other types of toxicity in RT or worsening in QoL [34, 35].

The QoL of patients in the study was evaluated at the beginning and end of RT treatment. Analyzing the results of both questionnaires we observed very few changes in QoL for the vast majority of domains evaluated when comparing patients from GHipo with GConv. This shows both a homogeneity between the groups in terms of QoL before starting RT treatment and that the type of protocol proposed does not significantly alter the QoL of the population studied. Although we can find in the literature studies such as that by Oba et al. (2021) that correlate a worsening in QoL as the number of RT sessions increases in a single group of patients receiving the same RT protocol[36]. We did not find works that compare the QoL in different RT protocols. Our results presented here were not intended to assess the worsening of quality of life before and after radiotherapy treatment within the same group, which would certainly imply a considerable deterioration in several domains, but rather to compare whether the type of radiotherapy protocol used implies in important differences in QoL at the end of treatment. In summary, our data show that the hypofractionated protocol did not worsen the QoL in this group, although it worsened oral ulceration.

The study has some limitations, such as the sample size, the protocol of the institution that did not include a microbiological diagnosis for candidiasis, and the absence of a dental service to accompany the patient throughout the treatment, which could have restricted information.

## Conclusion

This study aimed to evaluate the incidences of oral mucositis and candidiasis, and QoL in patients with HNC who underwent a hypofractionated RT protocol compared to a conventional RT protocol. Although mucositis worsened in patients treated with hypofractionated RT, QoL had not worsened for patients on this regimen. Clinical evaluation of candidiasis showed no difference between groups.

## Supplementary Information

**Additional file 1: Table 1S.** Evaluation of mucositis according to concurrent chemotherapy treatment.

## Data Availability

The dataset analyzed during the current study is available from the corresponding author on reasonable request.

## Abbreviations

COVID-19: Coronavirus disease 2019
GConv: Conventional group
GHipo: Hypofractionation group
HNC: Head and neck cancer
QoL: Quality of life
RT: Radiotherapy
SARS-CoV-2: Severe acute respiratory syndrome coronavirus 2
