# Supplementary material for: Evaluation of oral mucositis, candidiasis, and quality of life in patients with head and neck cancer treated with a hypofractionated or conventional radiotherapy protocol: a longitudinal, prospective, observational study
Source: Head Face Med. 2023 Mar 8;19:7. doi: 10.1186/s13005-023-00356-3 (PMC9992900; doi:10.1186/s13005-023-00356-3)
Supplement: Supplementary file 1 — Additional file 1: Table 1S. Evaluation of mucositis according to concurrent chemotherapy treatment. [file 13005_2023_356_MOESM1_ESM.docx]

Table 1S: Evaluation of mucositis according to concurrent chemotherapy treatment.

| Variables | Concurrent chemotherapy | | | |
| --- | --- | --- | --- | --- |
|  | No | Yes | Total | p-value |
| **Mucositis**  **(Intermediate evaluation)** | n (%) | n (%) | n (%) |  |
| No | 18 (62,1%) | 16 (43,2%) | 34 (51,5%) | 0,129^#^ |
| Yes  Total | 11 (37,9%)  29 (100%) | 21 (56,8%)  37 (100%) | 32 (48,5%)  66 (100%) |  |
| **(Final evaluation)** |  |  |  |  |
| No | 16 (55,2%) | 17 (45,9%) | 33 (50,0%) | 0,457^# #^ |
| Yes  Total | 13 (44,8%)  29(100%) | 20 (54,1%)  37 (100%) | 33 (50,0%)  66(100%) |  |
|  |  |  |  |  |
| **Mucositis, according to groups**  **(Intermediate evaluation)** | | | |  |
| **Ghipo** |  |  |  |  |
| No | 5 (55,6%) | 4 (28,6%) | 9 (39,1%) | 0,383^#^ |
| Yes | 4 (44,4%) | 10 (71,4%) | 14 (60,9%) |  |
| Total | 9 (100%) | 14 (100%) | 23 (100%) |  |
| **Gconv** |  |  |  |  |
| No | 13 (65,0%) | 12 (52,2%) | 25 (58,1%) | 0,395^#^ |
| Yes | 7 (35,0%) | 11 (47,8%) | 18 (41,9%) |  |
| Total | 20 (100%) | 23 (100%) | 43 (100%) |  |
| **(Final evaluation)** | | | |  |
| **Ghipo** |  |  |  |  |
| No | 4 (44,4%) | 3 (14,3%) | 6 (26,1%) | 0,162^# #^ |
| Yes | 5 (55,6%) | 12 (85,7%) | 17 (73,9%) |  |
| Total | 9 (100%) | 14 (100%) | 23 (100%) |  |
| **Gconv** |  |  |  |  |
| No | 12 (60,0%) | 15 (65,2%) | 27 (62,8%) | 0,721^#^ |
| Yes  Total | 8 (40,0%)  20 (100%) | 8 (34,8%)  23 (100%) | 16 (37,2%)  43 (100%) |  |
| ^#^ Chi-square test; ^# #^ Fisher's test | |  |  |  |
